# Studying Mixing in Non-Newtonian Blue Maize Flour Suspensions Using Color Analysis

**DOI:** 10.1371/journal.pone.0112954

**Published:** 2014-11-17

**Authors:** Grissel Trujillo-de Santiago, Cecilia Rojas-de Gante, Silverio García-Lara, Adriana Ballescá-Estrada, Mario Moisés Alvarez

**Affiliations:** 1 Centro de Biotecnología-FEMSA, Tecnológico de Monterrey, Monterrey, Nuevo León, México; 2 Centro de Investigación y Desarrollo de Proteínas (CIDPRO), Tecnológico de Monterrey, Monterrey, Nuevo León, México; 3 Departamento de Ingeniería en Biotecnología, Tecnológico de Monterrey, Tlalpan, Distrito Federal, México; University of Washington, United States of America

## Abstract

**Background:**

Non-Newtonian fluids occur in many relevant flow and mixing scenarios at the lab and industrial scale. The addition of acid or basic solutions to a non-Newtonian fluid is not an infrequent operation, particularly in Biotechnology applications where the pH of Non-Newtonian culture broths is usually regulated using this strategy.

**Methodology and Findings:**

We conducted mixing experiments in agitated vessels using Non-Newtonian blue maize flour suspensions. Acid or basic pulses were injected to reveal mixing patterns and flow structures and to follow their time evolution. No foreign pH indicator was used as blue maize flours naturally contain anthocyanins that act as a native, wide spectrum, pH indicator. We describe a novel method to quantitate mixedness and mixing evolution through Dynamic Color Analysis (DCA) in this system. Color readings corresponding to different times and locations within the mixing vessel were taken with a digital camera (or a colorimeter) and translated to the CIE*Lab* scale of colors. We use distances in the *Lab* space, a 3D color space, between a particular mixing state and the final mixing point to characterize segregation/mixing in the system.

**Conclusion and Relevance:**

Blue maize suspensions represent an adequate and flexible model to study mixing (and fluid mechanics in general) in Non-Newtonian suspensions using acid/base tracer injections. Simple strategies based on the evaluation of color distances in the CIE*Lab* space (or other scales such as HSB) can be adapted to characterize mixedness and mixing evolution in experiments using blue maize suspensions.

## Introduction

Mixing is one of the most common unit operations in the chemical engineering practice. However, the spectrum of techniques used and the mixing systems studied is still limited to relatively simple scenarios. The methods to characterize mixing are mostly focused in Newtonian liquid-liquid systems, but the vast number of techniques currently available to evaluate mixing quality or extent are restricted to transparent or nearly transparent vessels and fluid systems (water, glycerin solutions, diluted suspensions, solutions of CMC in water, Carbopol 940).

More complex and realistic mixing scenarios have received only modest attention in the fluid mechanics and physics literature. One specific scenario is the mixing of heavy suspensions (suspensions where the concentration of solids is higher than 5% w/w), an operation that occurs in diverse lab and industrial scale settings in many applications related to food technology [1]–[3], polymer processing [4]–[5], and biotechnology [6]–[9], among others. In many of these experimentally relevant suspensions, Non-Newtonian behavior is observed [6]–[11].

Only a limited number of papers have addressed the mixing in Non-Newtonian systems, focusing mainly on solutions [12]–[15]. Even fewer studies have addressed mixing of realistic Non-Newtonian suspensions. At high solid densities, the opaqueness produced by the particles presents an obstacle to the use of classical visualization techniques. Recently, 2D and 3D tomography was applied to the study of mixing in non-transparent fluids [12]. Laser Sheet Image Analysis (LSIA), a sort of laser-based tomography, was formally introduced as a technique to study the dynamics of dilute particle suspensions [16]. Positron emission particle tracking (PEPT) has been used to track individual particles in heavy slurries [17], producing valuable (Lagrangian type) information on the dynamics of heavy suspensions. However, the implementation of tomography and PEPT techniques demands special infrastructure not widely available in the common fluid mechanics lab. Besides, in a highly viscous system or a suspension, the adequate dispersion of the indicator might be an issue in itself. In particular, only a few studies refer the use of tracer techniques to investigate mixing in heavy suspensions; that is, with solids content above 5% [17].

**Figure 1:**
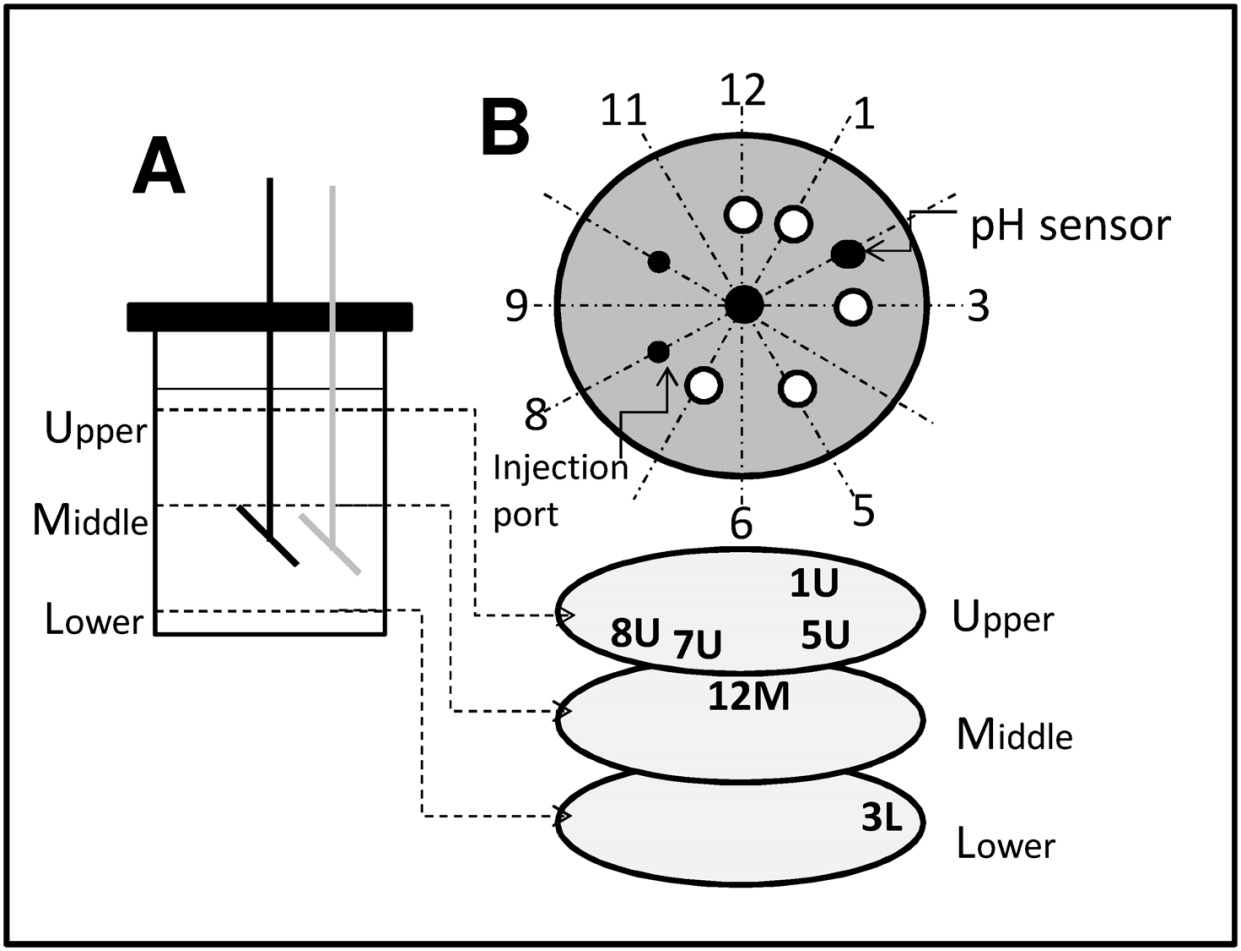
Scheme of the experimental system. (A) A stirred tank, equipped with a 45° inclined disk impeller, was used to agitate non-Newtonian blue maize suspensions. Three different horizontal planes [upper-plane (U), mid-plane (M), and lower-plane (L)] were defined within the tank for tracer injections. For some of our experiments, the impeller was placed in an eccentric position (indicated in gray). (B) The tank lid was modified to allow for additions of acid/base solutions at different angular positions (12 o’clock, 1 o’clock, etc.).

**Figure 2:**
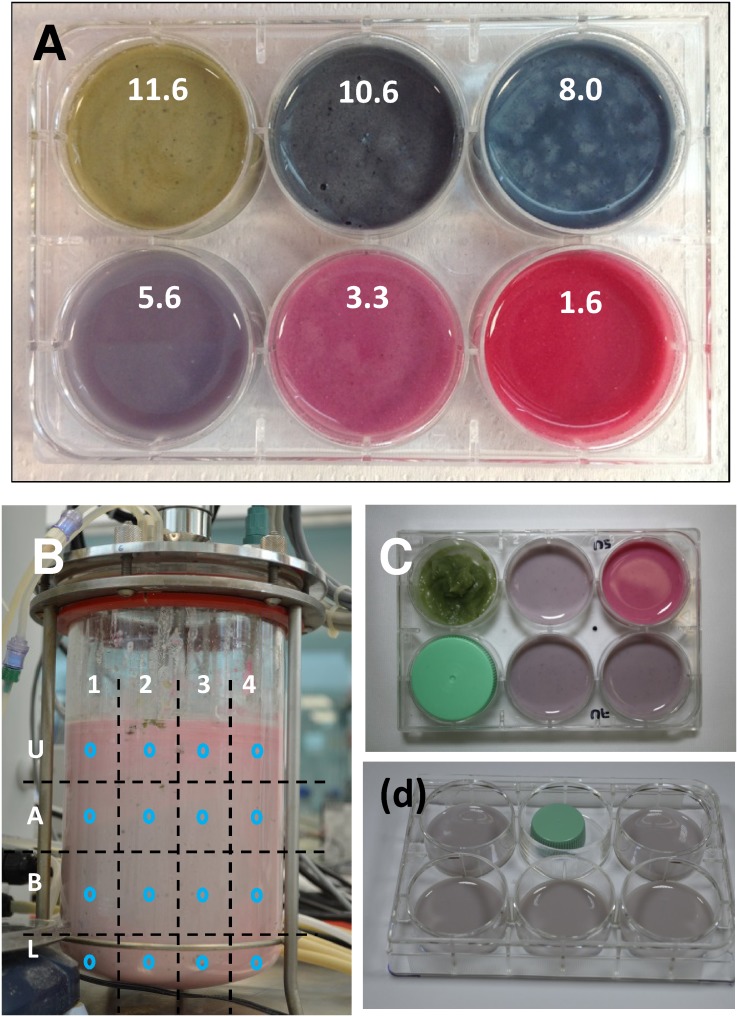
Following mixing through color changes in blue-maize suspensions. (A) In blue maize suspension, the color varies significantly as a function of pH due to the presence of native anthocyanins that act as a natural wide spectrum pH indicator. (B) The evolution of mixing of a blue maize flour suspension in a stirred tank was followed by addition of a basic injection into an initially acidic condition. Frontal photographic images were taken at different time points of the mixing process. Each image was divided into sixteen sections (U1 to L4) and the color in the CIE*Lab* scale was determined by image analysis at each of the center points (indicated by blue circles). (C) Samples corresponding to different tank locations and times of agitation were dispensed in 6-well culture plates for color analysis using digital photography or colorimetric readings with a portable colorimeter. Reproducibility of the color readings among different plates can be validated by including a color standard in each plate (in this case, a circular plastic object of uniform color). (D) The experimental error associated with lighting heterogeneity at different well positions was estimated by placing the same sample in different wells.

Here, we use blue maize flour suspensions stirred in stirred tanks with a simplified geometry (no baffles, disc impellers; see Figure 1) to illustrate the existence of severe mixing non-idealities in non-Newtonian systems. As a secondary objective, we propose the use of blue maize suspensions as a useful and flexible model for the study of mixing of Non-Newtonian suspensions. Blue maize is a corn variety native to México [18], [19]. Blue maize flour suspensions exhibit Non-Newtonian behavior (Figure 2), and are convenient for studying mixing in heavy suspension experiments because the anthocyanins naturally present in blue maize kernels [18], [20], [21] serve as a natural pH indicator that undergoes drastic color changes in a wide range of pH values. Therefore, the simple injection of acid or base solutions allows visualization of flow patterns and the qualitative and quantitative characterization of mixing.

Industrial processes in which acid or basic injections are added to a non-Newtonian fluid, are not infrequently encountered, particularly in Biotechnology applications where the pH of non-Newtonian culture broths is usually regulated by the addition of acid/base solutions [8], [11], [22], or where the bioreaction itself releases acid into a non-Newtonian fluid, further modifying its rheology [23], [24].

## Results and Discussion

### Blue maize suspensions as a complex fluid model for mixing experiment

Blue maize suspensions allow performing flow visualization experiments without the need to add a foreign pH indicator. The anthocyanins naturally present in blue maize flour [20], [21] respond to changes in pH by displaying a wide range of colors (see Figure 2a). At low pH values, blue maize suspensions exhibit a pink color. Progressively, as pH is increased, this color transitions to magenta, pink, violet, blue, blue-greenish, and green.

The presence of this intrinsic and wide range pH indicator has important practical advantages in the laboratory. With a few exceptions [25], [26], most available pH indicators exhibit a narrow range of color change, from three to five pH units [26], [27], which limits the window of visualization for the occurrence of the acid-base reaction. The simultaneous use of two or more pH indicators has also been suggested to amplify the span of pH sensing [28]. However, in practice, the dispersion of a foreign indicator in a non-Newtonian (or Newtonian but highly viscous) fluid is, in itself, a non-trivial mixing problem.

**Figure 3:**
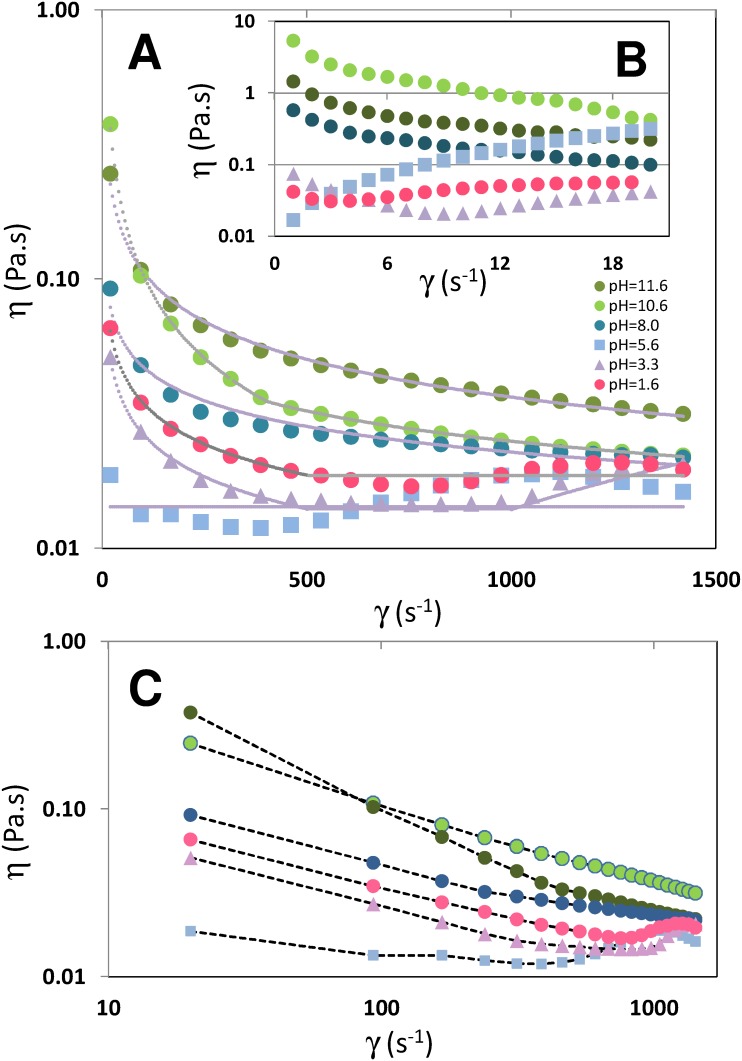
Blue maize flour suspensions exhibit different rheological behavior at different pH values. (A) Plot of apparent viscosity versus shear rate (in the range from 250 to 1500 s^−1^) for blue maize suspensions prepared at different pH values. Gray dotted lines correspond to power-law fits to experimental data based on the Ostwald-de Waele model [η = K (γ)^n–1^] using the parameter values reported in Table 1. (B) Plot of apparent viscosity versus shear rate (in the range from 250 to 1500 s^−1^) for blue maize suspensions prepared at different pH values. (C) Log-log version of the plot of apparent viscosity versus shear rate (in the range from 250 to 1500 s^−1^) for blue maize suspensions prepared at different pH values. Straight dotted lines have been used to connect the experimental data points.

The non-Newtonian behavior of flour suspensions has been characterized in the context of food engineering applications [29]–[31]. We conducted determinations of shear stress and apparent viscosity at different shear rate values for the blue maize flour suspensions used in our experiments using an automatic Rheometer (Physica MCR Anton Paar, Austria). Figure 3a shows the apparent viscosity values in the range of shear rates from 20 to 1520 s^−1^; we observe non-Newtonian behavior across various pH values (Figure 3a, 3c). At low shear rate values (Figure 3b), except under very acidic conditions (3.3> pH >1.6), suspensions displayed an evident non-Newtonian behavior with apparent viscosities varying drastically as a function of shear rate.

The rheology of our blue maize suspensions at different pH values can be described approximately by a simple Ostwald-de Waele power-law model (see Equation 1).
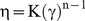
(1)Here η is the apparent viscosity at a given shear stress and pH condition, γ is the shear rate, K is a flow consistency index, and n is the flow behavior index. In Table 1, we present best-fit values for K and n for our blue maize suspensions calculated from linear regressions of the type ln η = ln K+(n–1) ln (γ). For suspensions at pH = 5.6, a Newtonian behavior is observed. For higher pH values (8.0, 10.6, 11.6) a clear non-Newtonian shear thinning behavior is evident (n<1). A striking flow behavior is observed in suspensions at pH = 3.3 and 1.6, at which the flow transits from shear thinning (at low shear rates) to shear thickening (at high shear rates). To model this transition without recourse to a more complex model, we simply provide piece-wise valid values for K and n.

**Table 1:** Proposed set of values for K and n for the Ostwald-de Waele power-law equation [η = K (γ)^n–1^] to model the rheology of blue maize suspensions at different pH values.

pH	shear range (s^−1^)	n–1	K (Pa s^n^)	n
11.6	20–1420	–0.4660	0.9102	0.5340
10.6	20–500	–0.7772	3.7109	0.2228
10.6	500–1420	–0.3735	0.3293	0.6265
8.0	20–1420	–0.3165	0.2025	0.6835
5.6	20–1420	0	0.0142	1.0000
3.3	20–500	–0.3979	0.1657	0.6021
3.3	500–1000	0	0.0140	1.0000
3.3	1000–1420	1.1348	5.52E-6	2.1348
1.6	20–500	–0.3823	2.01E-1	0.6177
1.6	500–1420	0	0.01865	1.0000

K and n were calculated from linear regressions of the type ln η = ln K+(n–1) ln (γ).

One question is how significant are these variations of apparent viscosities in the context of a stirred tank system. In a stirred tank, the range of shear rates spans four to five orders of magnitude, even in Newtonian systems [32], [33]. Based on theoretical arguments, Sánchez-Pérez et al. [34] recently proposed the expression γ∞ N ^3/2^, where N is the agitation speed in RPM, to establish a general relationship between agitation speed and maximum shear rate in turbulent stirred tanks. The authors also observed that the expression γ = 33.1 N^1.4^, consistent with their theoretical derivation, correlates well with data previously calculated [35] for stirred vessels using computational fluid dynamics. In our experimental tank system, we agitated at N = 1000 RPM (16 rev/s). Therefore, assuming a fully turbulent regime in the impeller zone, the maximum shear rate value will marginally exceed 2000 s^−1^, and the average shear rate value will be in the neighborhood of 20 s^−1^. However, areas of low shear (∼2 s^−1^) could be found near the tank walls and in low circulation areas close to the tank bottom or the tank surface. Based on normalized distributions of shear rates reported for stirred tanks [33], approximately 10% of the tank volume experiences shear values below 5–6 s^−1^. For a non-Newtonian system this has profound implications, and the higher apparent viscosities observed at low shear values (Figure 3b) represent an added complexity to mixing. Zero-shear-viscosity (in Pa.s), calculated from Figure 3b, are 1.737, 7.550, 0.702, 0.019, 0.086, 0.054, at pH = 11.6, pH = 10.6, pH = 8.0, pH = 5.6, pH = 3.3, and pH = 1.6, respectively.

In the following sections, we discuss mixing experiments conducted in blue maize suspensions of nearly 50% solids. First, we describe the use of blue maize suspensions as a fluid model for qualitative study of mixing in different stirred tank configurations. Then, we demonstrate the use of simple techniques to quantitate mixing evolution based on the analysis of color changes (digital color analysis; DCA), associated with pH changes, during acid/base injection/excursion experiments. Two examples of the use of DCA techniques are provided. The first example employs a simple, non-intrusive strategy that is suitable for transparent vessels. We analyze a time-series of photographic images of the exterior of the entire stirred tank system. The second application case uses DCA to analyze images from samples taken at different tank locations at different agitation times. This second example extends the use of DCA to non-transparent agitation/blending vessels.

### Mixing visualization in blue maize suspensions

Mixing dynamics in non-Newtonian systems can become highly complex. Our experimental observations suggest that the mixing performance of non-Newtonian systems is extremely sensitive to some geometrical and operational parameters. The location of the stirring axes, the location of the point of injection and the starting condition (acidic or basic) are important considerations that define mixing performance.

**Figure 4:**
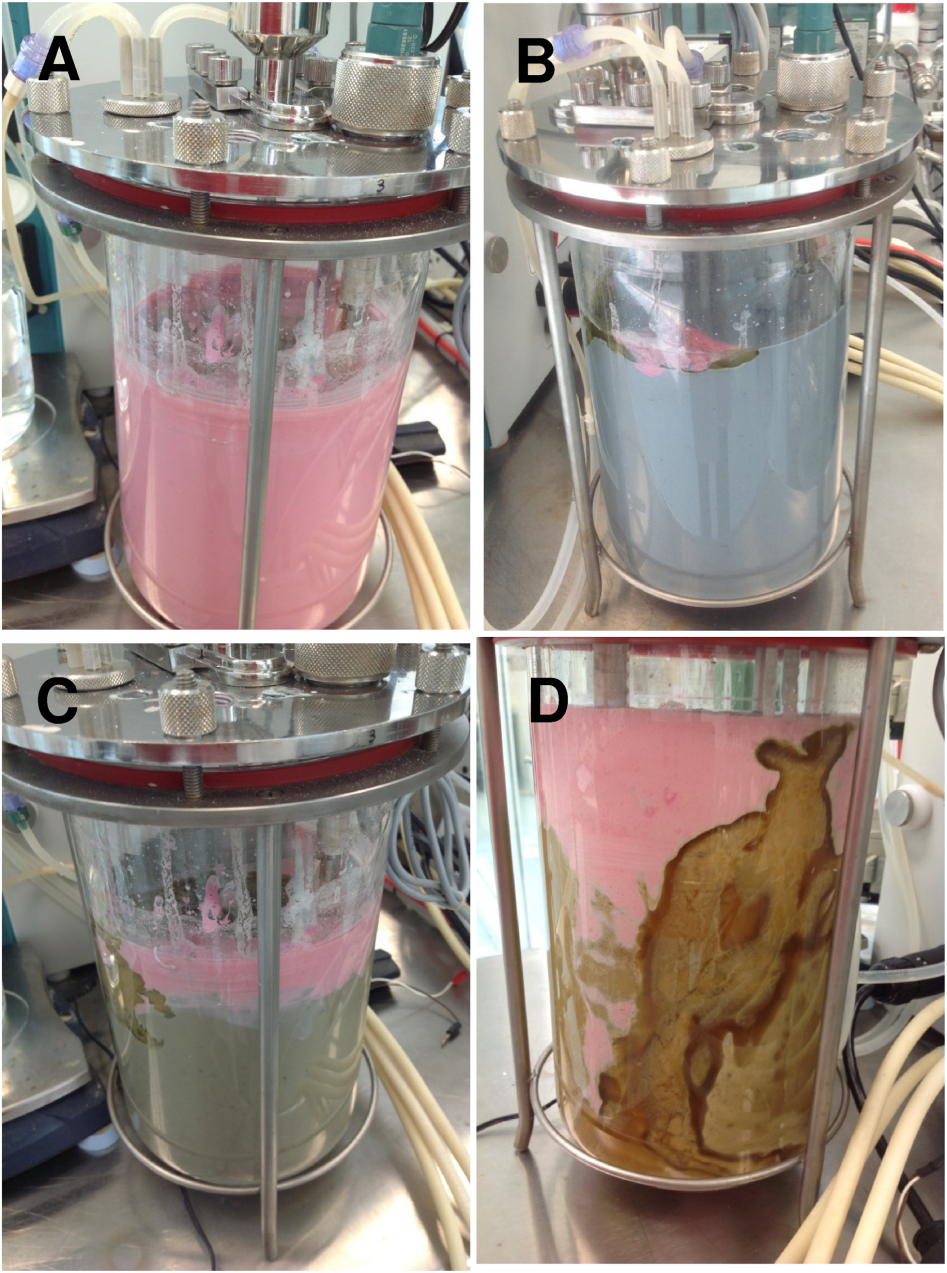
Different mixing states in a stirred tanks containing blue maize non-Newtonian flour suspensions. (A) In a tank stirred by an eccentrically located inclined disc impeller, a subsurface acid injection was efficiently dispersed to achieve homogeneity. (B) Severe top segregation is evident following a subsurface base injection in a concentrically agitated system. The inadequate selection of the point of addition of a concentrated basic injection can lead to the creation of (C) stagnant zones where alkaline conditions prevail, causing high viscosity conditions and (D) further obstructing effective mixing.

**Figure 5:**
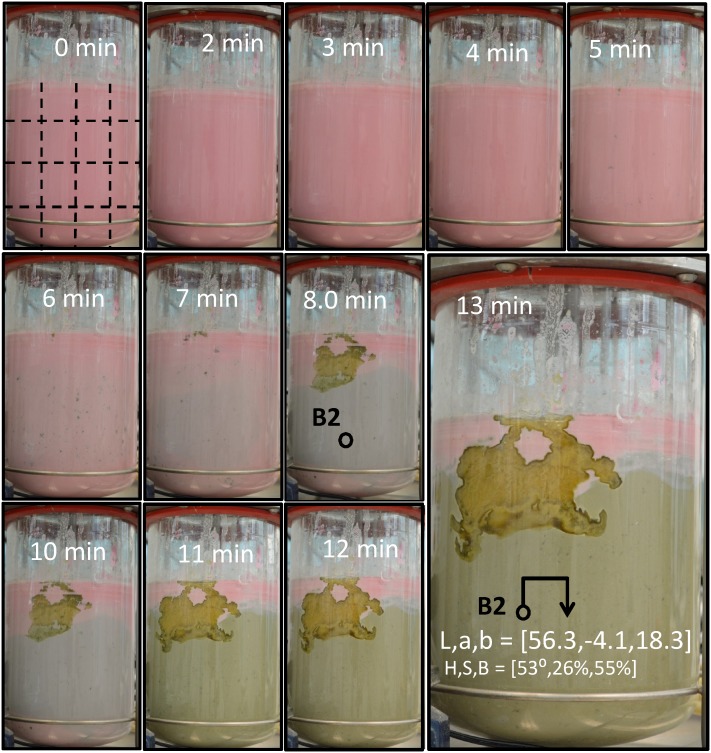
Blue maize suspensions allow the performance of acid-base experiments to visually evaluate the progression of mixing in transparent agitation systems. Mixing experiment that shows evolution in a conventional stirred tank geometry from an initially homogeneous acidic state (t = 0) towards a final process state (t = 13.0 min), in which segregation still prevails (particularly top-bottom segregation). Readings of color, in the CIE*Lab* and the HSB system, are indicated for location B2.

In our experiments, eccentric stirred tank configurations outperform concentric ones, particularly when the aspect ratio is higher than 1.2. Figure 4 illustrate these findings, showing different mixing conditions or states in a stirred tank containing a blue maize non-Newtonian flour suspension. In Figure 4a, which depicts a tank stirred by an eccentrically located inclined disc impeller [36], [37], a subsurface acid injection was efficiently dispersed to achieve a practically (at least visually) homogeneous condition in less than 5 minutes. In Figure 4b, we show the final state of mixing of a similar experiment. This time, the initial pH was acidic, and a basic solution (i.e., NaOH 1N) was administered at the fluid surface. Even when most of the system has reached a basic pH, segregated acidic areas prevail at the liquid surface. A frequently observed mixing problem in conventional stirred tank geometries is the presence of segregated or low circulation regions at the upper section of the tank. A recent contribution [38] demonstrates that Reynolds numbers above 300,000 (based on the impeller diameter and speed) are needed to assure fully turbulent regimes in the upper third section of typical stirred tank configurations. In non-Newtonian system, this mixing pathology is even more evident under certain conditions. For example, when a concentrically agitated system is used, a layer of acidic material is still evident in the upper section of the tank several minutes after a subsurface basic injection into an initially acid environment (Figure 4c). Figure 5 presents a sequence of images corresponding to this experiment, in which an initially acidic blue maize flour suspension is agitated in a conventional tank after a basic set point has been established. The tank is equipped with a concentrically located radial impeller rotating clockwise at 1000 RPM; the height/diameter ratio (H/D) is 1.15, and the impeller is located at approximately 1/4 H. This experiment visually illustrates the progression of mixing in a concentrically agitated tank, from an initial homogeneous condition, to a final state that shows top-bottom segregation. In this case, although the reaction is instantaneous, the rheology of the system imposes conditions that slow the advance of the acid/base reaction, and the mixing process becomes limiting, finally determining the rate of the overall process. Images show, qualitatively, that concentric systems with this geometry (H/D between 1.1 and 1.3 and one agitator) retain certain top-bottom segregation, even after extended periods of agitation.

The blue maize suspension system can be particularly useful for conducting experiments to diagnose poor or ideal injection locations. At pH values above 8.0, the viscosity of blue maize suspensions increases significantly and abruptly, reducing flow and obstructing mixing even more. Therefore, in experiments where a basic solution is dispensed through a point of injection in a low circulation zone, stagnant regions will develop. These regions can be easily detected by the development of a green color, characteristic of basic pH values (see Figure 2a and 2c). In our stirred tank system, the inadequate selection of the point of addition of a concentrated basic solution, even in eccentric configurations, can lead to the creation of these stagnant zones where alkaline conditions prevail, causing high viscosity conditions and further obstructing effective mixing (Figure 4d). The gelatinization induced in corn flour and starch suspensions by alkaline conditions has been studied in detail [39].

### Following mixing dynamics in a color space using images

Here, we introduce a simple methodology for quantitation of the state of mixedness and the mixing dynamics through the analysis of color changes in the CIE*Lab* scale, one of the color scales normally used for image analysis and color description applications [40], [41]. The use of Digital Color Analysis (DCA), utilizing colors or the digital information embedded in colors, has been suggested before as a tool for quantifying chromatic changes [42]. Here we show how a sequence of images can be analyzed using simple DCA methods to diagnose mixing evolution and mixing extent. For example, a reference grid can be used (see Figure 2b) to define a number of sections within every image in Figure 5. At every region, a series of sampling points can be defined.

**Figure 6:**
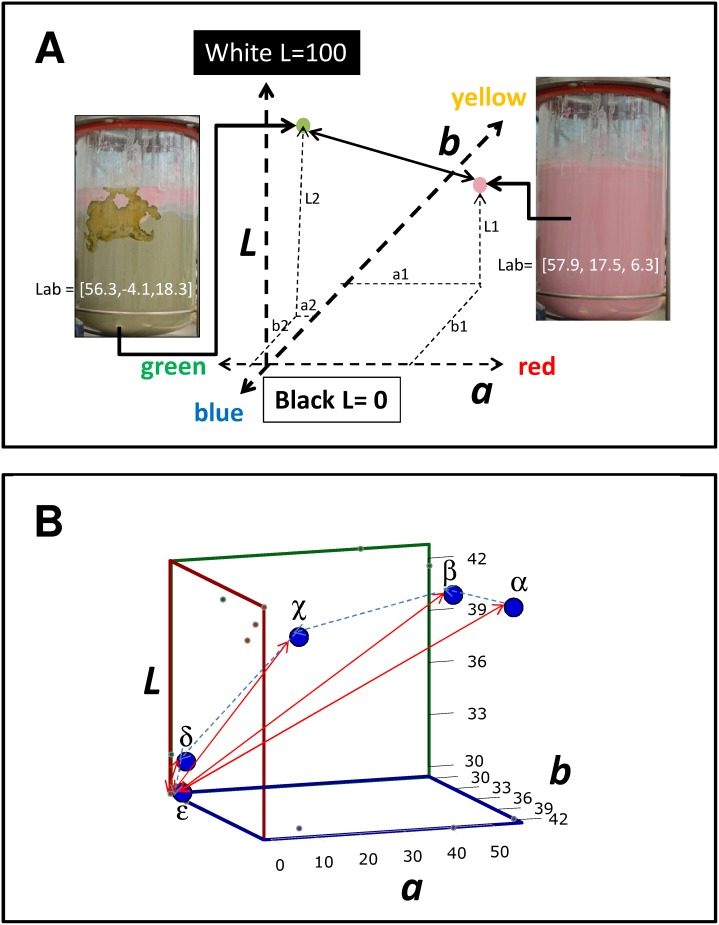
The estimation of distance between two points in the *Lab* scale of colors provides a parameter for characterizing the state of mixing in a stirred system. (A) The CIE*Lab* scale represents a color in a three-coordinate system in which L is associated with luminosity, ranging from 0 for black to +100 for white. The a and b values define a plane of colors,, where a ranges from negative to positive values (green to red) and b ranges negative to positive values (blue to yellow). The difference between two colors can be determined by calculating the distance between them in this 3D space. (B) Progression of pH values during a typical acid or base injection experiment in blue maize flour suspensions (from pH 10.55 to 1.6). Values are plotted in a 3D space; they describe a mixing process trajectory (dashed blue line). The distance between each pH value in the trajectory and the final process point (indicated with red arrows) can be calculated. The values of distances with respect to this final point consistently decrease.



Let us consider that the center-point within each zone will be used as a “sample” location to determine color according to the *Lab* scale. In this system, each color is characterized by three values, *L*, *a*, and *b*. The *L* value is associated to luminosity, ranging from 0 for black, and +100, for white. The *a* and *b* values define a plane of colors, as shown in Figure 6a, where *a* ranges from negative to positive values (green to red) and b ranges from negative to positive values (blue to yellow). Therefore, for each sampling point in each image of Figure 5, the color can be characterized by the *L*, *a*, and *b* coordinates that define a unique point in the *Lab* color space. One could estimate the deviation of a particular state of mixing (at a particular location and time) from a final mixing point or an “ideal mixing” state (presumably the final condition of complete mixing), by evaluating the differences in colors between the two states. Conceptually, one way of doing this is by evaluating the distances between the two corresponding points in the *Lab* coordinate system (Figure 6a). The distance in the *Lab* space, defined by a straight line connecting both points, can be calculated by equation 2.

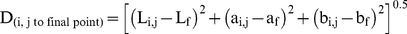
(2)Here, D_(i,j to final point)_ is the distance, in the *Lab* space, of the points defined by the *Lab* coordinates of the sample taken at time *i* and location *j* (L_i,j_, a_i,j_, b_i,j_) and a sample representative of the final mixing state (L_f_, a_f_, b_f_).

**Figure 7:**
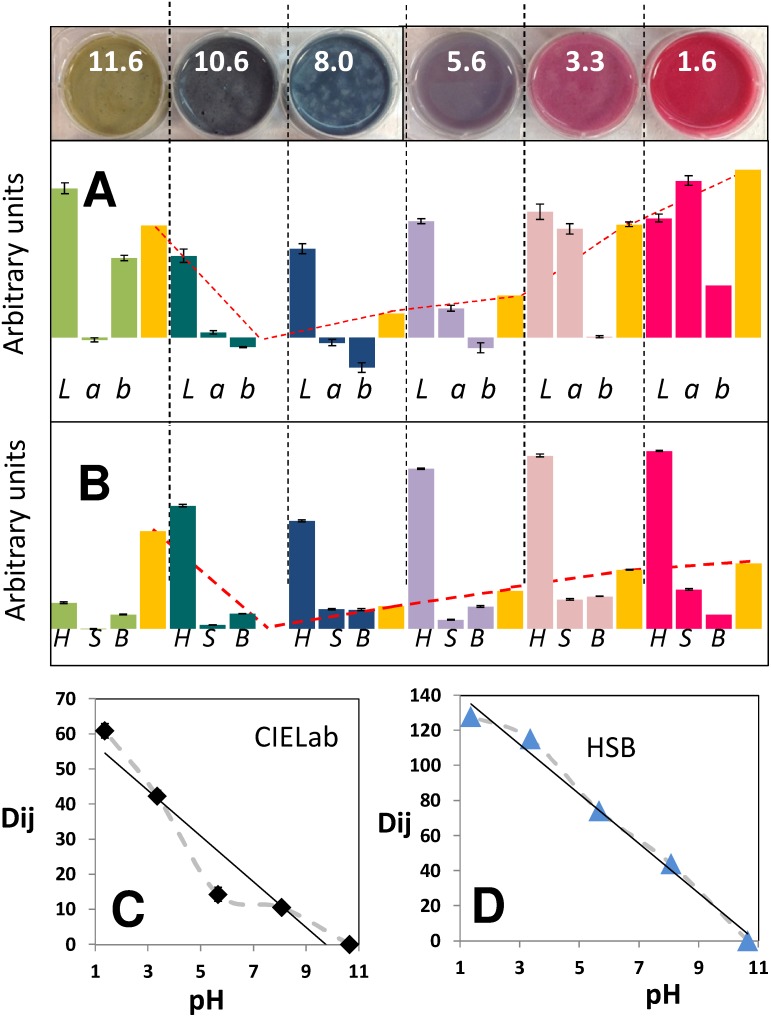
Variability of color readings at different pH values in blue maize flour samples. (A) The average value of the three color components of the CIE*Lab* scale (L, a, and b) are presented for six color determinations at each pH value. Bars are colored according to the pH value, resembling their tone in the maize suspension. Error bars indicate the standard deviation of six determinations. Yellow bars indicate the average color distance (D_i,j_), as calculated from equation 2, with respect to the *Lab* values corresponding to the sample at pH = 10.6. A dotted red line indicates the decreasing trend in D_i,j_ values when the sample at pH = 10.6 is taken as a reference (or final point). (B) The same determination was conducted using the HSB scale of colors. Yellow bars indicate the average color distance (D_i,j_), as calculated from equation 2, with respect to the *Lab* values corresponding to the sample at pH = 10.6. A dotted red line indicates the decreasing trend in D_i,j_ values when the sample at pH = 10.6 is taken as a reference (or final point). (C) Plot of D_i,j_ values in the CIE*Lab* scale of colors versus pH for a pH trajectory from pH 1.3 to 10.6. (D) Plot of D_i,j_ values in the HSB color system versus pH for a pH trajectory from pH 1.3 to 10.6.

The use of distances between a particular point and a final mixing point as an indicator of deviation from homogeneity would be valid only if that distance consistently decreases as the system becomes more homogeneous. This condition should be validated for each indicator system. In the particular case of the anthocyanins naturally present in blue maize suspensions, this condition is satisfied for a wide range of pH values. Figure 6b shows the progression of colors in a blue maize suspension as pH values increase from an extremely acidic condition (pH = 1.6; point α) to an extremely basic condition (pH = 11.6; point φ). For each of the six pH values in this set (α, β, χ, δ, ε, φ), a color can be defined in the *Lab* scale, corresponding to a particular point in the 3D *Lab* color space (Figure 7b). Let us now define a pH trajectory from pH = 1.6 to pH = 10.6 (from point α to point ε). For each of the five points in this trajectory, a value of the distance with respect to the final point can be calculated (D_i,ε_). For this pH range of variation, the vector (D_α,ε_, D_β,ε_, D_χ,ε_, D_δ,ε_, D_ε,ε_) is (60.85, 42.27, 14.26, 10.59, 0). The value of distance in colors decreases as the system moves from pHα to pHε. Therefore, in a pH excursion experiment, where the initial condition is in this range of pH values, and the final point is in the neighborhood of 10.6, the value D_i,j_ would be a valid indicator of heterogeneity. A similar analysis can be formulated considering point φ as the final mixing state for a mixing trajectory. In the range of pH = 1.6 to pH = 11.6, the value of the vector (D_α,φ_, D_β,φ_, D_χ,φ_, D_δ,φ_, D_ε,φ_) is (58.85, 50.95, 36.41, 45.22, 37.69, 0), and the requirement for decreasing D_i,j_ values is not fulfilled. Therefore, our analysis suggests that in this system (blue maize suspensions), pH excursion experiments to characterize mixing using DCA should be conducted in the pH range between 1.6 and 10.6. Starting at pH 1.6 and ending in 10.6 (or *vice versa*) takes advantage of the widest pH span possible. Evidently, the reliability of this strategy for characterizing mixing will also depend on the robustness of the determination of color. External factors such as uneven illumination or intrinsic variations in tone (particularly in a suspension) may affect the determination of color for a particular sample.

**Figure 8:**
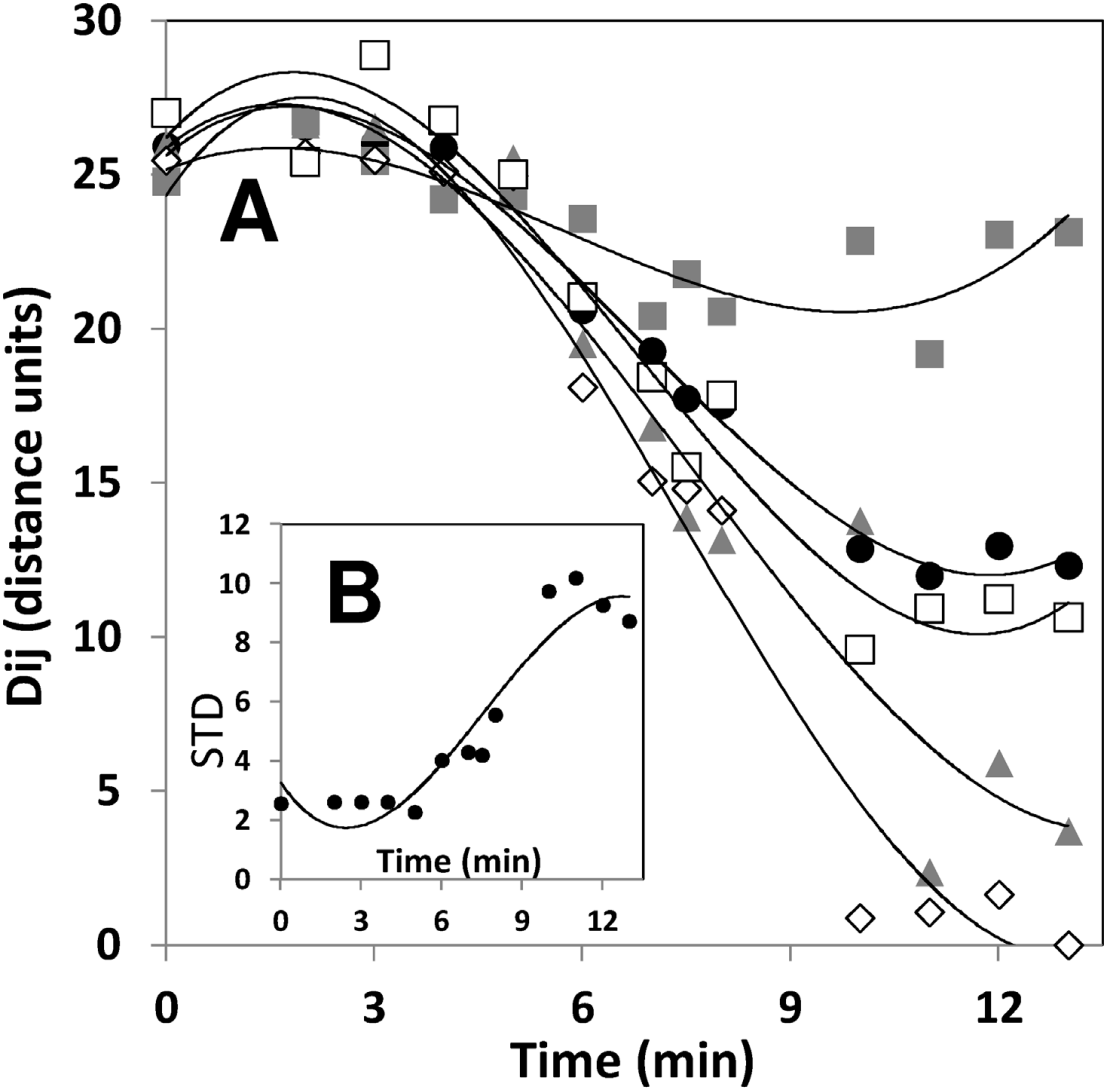
Evaluation of mixing progression using distances between colors. (A) Evolution of the distance in color, based on the CIE*Lab* color space, with respect to the final mixing state (D_i,j_) at different tank locations for the experiment in Figure 5: at U2 (▪); at A2 (▴); B2 (◊); L2 (□). The average distance with respect to the final point, considering all sampling points, at different agitation times is also presented (•). Lines indicate polynomial fits to data. (B) Time evolution of the standard deviation of all the D_i,j_ values (corresponding to the same time point); a direct indicator of the degree of heterogeneity in the mixing conditions within the vessel. Lines indicate polynomial fits to data.

We conducted a simple experiment to validate the robustness of color estimations in the CIE*Lab* scale in samples of blue maize suspensions with different pH values in the range from 11.6 to 1.6. Samples were dispensed in cell culture wells. A digital photograph was taken under homogeneous illumination conditions under indirect white light. The picture was loaded into the image analysis application Color Companion 4.0 for iPad. At each well, corresponding to each pH value, multiple readings of color were conducted (in the *Lab* scale) and the average and standard deviation of each color component (*L, a*, and *b*) was calculated. In addition, for each color reading, the distance in color (D_i,j_) with respect to the average color corresponding to pH = 10.6 (assumed arbitrarily as a final point or reference point) was calculated using equation 2. The corresponding analysis is presented in Figure 8. The standard deviation of each component of each color is relatively low compared to variations due to pH. In addition, the standard deviation of D_i,j_ for each particular color determination is also relatively low compared to the D_i,j_ variation related to actual changes in pH. Moreover, in agreement with the analysis presented in Figure 7, the value of the distance (D_i,j_) consistently decreases in the range from pH = 1.6 to 10.6.

When designing pH excursion experiments in this pH range, the injection location [36], [43] and the direction of the pH trajectory are important considerations. The viscosity of blue maize suspensions increases substantially at high pH values (see Figure 3b and c). A poor selection of the point of injection of a basic pulse might lead to the creation of a basic locus that would further obstruct the tracer dispersion and the overall mixing process. On the other hand, to initiate a mixing experiment from a basic condition, injecting an acid pulse will demand higher agitation rates due to the higher viscosity of the initial condition.

### Feasibility of use of other color systems: RGB and HSB

We have explored the feasibility of using other scales of color to perform DCA in blue maize flour suspension mixing experiments. In particular, we considered the use of the RGB system (widely used in digital devices, computer screens, and Photoshop, among others) and the HSB scale (preferred in the context of art). In the range of pH from 1.6 (starting point) to 10.6 (final mixing state), the HSB color space behaves similarly to CIE*Lab*. The distance D_i,j_ decreases as pH decreases in this range for both the CIE*Lab* and the HSB system (Figure 7; Figure 8a). This is not the case if RGB is used: in the pH trajectory from 1.6 to 10.6, the D_i,j_ value does not always decrease as the mixing process advances. Indeed, our results suggest that the relationship D_i,j_ vs. pH is more linear (for this pH range of pH and color) in the HSB space of color than in the CIE*Lab* space (compare Figure 7c and 7d).

Although the strategy presented here also works effectively in HSB, in this contribution we mainly use the CiE*Lab* system to illustrate the analysis. The literature refers to several attributes/advantages of the CIE*Lab* space of color. For example, it includes all perceivable colors, which means that its gamut exceeds those of other color models (i.e., the RGB space used by ProPhoto includes about 90% all perceivable colors). The CIE*Lab* color space is also considered more perceptually uniform compared to other systems, meaning that a change of the same amount in a color value should produce a change of about the same visual importance. In addition, the CIE*Lab* model is considered to be device independent and it has been more frequently used than HSB has in the scientific literature.

### Example of application: following mixing in transparent vessels

Let us consider a sample taken at a time *i* and a location *j* (for example, t = 8 min and location B3 in Figure 5). The color at this sample point in the *Lab* scale, as determined by analysis using the Color Companion 4.0 for iPad application, was L_i,j_ = 59.2, a_i,j_ = 0.4, b_i,j_ = 4.9. By visual inspection, we can approximate a final state for the mixing process. For example, here, the average of the *Lab* values at the final time point (t = 13 min; Figure 5) at the location B2 (i.e., L_f_ = 56.3, a_f_ = –4.1, b_f_ = 18.3) was considered to approximate a desirable well-mixed condition. For these two *Lab* value points, the value of the D_8min,B3_ to final point is 14.4. For the set of images in Figure 5, we calculated the distance in the *Lab* color space (D_i,j_), as defined by equation 2, for each one of sixteen reading positions (U1 to L4) and 13 time points considered in the experiment. D_i,j_ was calculated with respect to the *Lab* color values at the location B2, at t = 13 min. The average distance of these D_i,j_ values is physically related to the global deviation of the system with respect to the final state of mixedness (Figure 8a). In addition, the standard deviation of all D_i,j_ values, corresponding to the same time point, is a direct indicator of the degree of heterogeneity in the mixing conditions within the vessel. Consistent with a simple visual analysis from the images, the reader will observe that, in this particular experiment, the system approaches a final state of mixedness in which segregation (indicated by the STD value) is more prevalent than at the initial condition (Figure 8b).

### Extension to non-transparent vessels

This strategy for mixing analysis based on DCA can be extended to non-transparent vessels. Let us consider a scenario in which the initial pH of the suspension is set to 6.5 by slow addition of 3N HCl through the port located at 8 o’clock (injection location 8U; see Figure 1). Then, a pH target point of 8.5 was set, and the control system was activated to deliver small pulses of a basic solution (1N NaOH solution) until the new pH set point were achieved. This time, the impeller axis has been displaced to an eccentric position (E = 0.45). Off-centered agitated stirred tanks have been described as an effective alternative for mixing in the laminar regime [36], [37], [44], [45]. Here we use eccentricity to improve mixing performance in our blue maize flour suspension system. We followed the evolution of mixing in this experiment during several hours of agitation. Again, the color of the suspension varies as the pH progressively changes. At five different tank locations (in this example 12M, 5U, 3L, 1U, 7U), we took 3 mL samples of the suspension and dispensed them in the wells of commercial 6-well culture plates, as shown in Figure 2c and 2d. The color of each of these samples was evaluated using a colorimeter (Chroma Meter CR-300 from Minolta, NJ, USA). Although the measurement can be taken from above or below each well, best results are obtained by placing the colorimeter underneath the samples, directly in contact with the bottom surface of each well.

**Figure 9:**
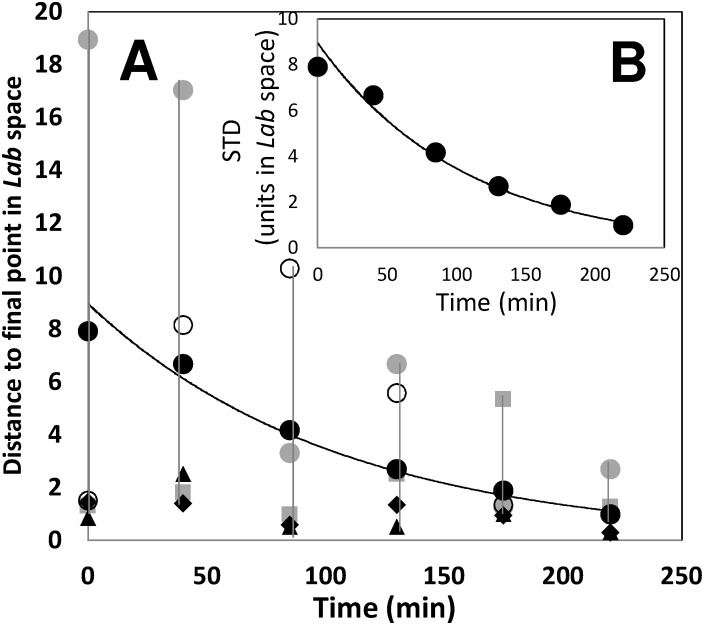
Quantitation of mixing through the concept of distances between mixedness states in the *Lab* color space. (A) Evolution of the average distance (D_i,j_) with respect to an ideal mixing state condition for five different sampling locations defined within the tank volume (see Figure 2): 7U (•); 1U (▪); 12M (▴); 5U (○); and 3L (♦). The average distance with respect to the final point is marked with black circles (•); a second order polynomial fitting is shown (solid curve). (B) Evolution of the standard deviation from an initially segregated condition to a homogeneous final state (•). A second order polynomial fitting is shown (solid curve).

Once more, mixing can be followed by evaluating the distances between mixing states in the *Lab* coordinate system. Let us consider a sample taken at a time *i* and a location *j* (for example, t = 40 min and location 12M in Figure 1b). The color of this sample in the *Lab* scale, as determined by the colorimeter reading, was L_i,j_ = 24.63, a_i,j_ = –0.61, b_i,j_ = 2.78. At the final point of the experiment, which approximates a well-mixed condition, the corresponding average *Lab* vector (constructed from the average value of *L, a,* and *b* for all five sampling locations at t = 13 min) was L_f_ = 22.992, a_f_ = –1.398, b_f_ = 1.05. For these two points, the distance in the *Lab* space, defined by a straight line connecting both points, can be calculated by equation 2. Here, D_(i,j to final point)_ is the distance, in the *Lab* space, of the points defined by the *Lab* coordinates of the sample taken at time *i* and location *j* (L_i,j_, a_i,j_, b_i,j_) and a sample representative of the final mixing state (L_f_, a_f_, b_f_). For the case under consideration, the value of the D_40min,12M to final point_ is 2.5094. For this very same time point, the other distance values in the *Lab* space, corresponding to other sampling points are D_40min7U to final point_ is 17.022, D_40min,1U to final point_ is 1.7953, D_40min,5U to final point_ is 8.1427, and D_40min,3L to final point_ is 1.3766. The average distance of these distance values is physically related to the global deviation of the system with respect to the final state of mixedness (Figure 9a). In addition, the standard deviation of these values, corresponding to the same time point, is a direct indicator of the degree of heterogeneity in the mixing conditions within the vessel (Figure 9b).

Our interest resides in the experimental zone of high density slurries. Within this region, the trend of diminishing D_i,j_ as pH varies from an acidic condition to a basic set point (i.e. pH 10.6) still holds, but the slope is slightly different for slurries of different concentrations. The slope variation is mainly based on variations in illumination. In the CIE*Lab* space, the intensity or luminosity of a particular color is mostly related to the L value. In general, at higher solid concentrations, the L value decreases for all pH values, while a and b are weaker functions of the concentration of solids in the slurry.

### Blue maize suspensions as a model: discussion on advantages and limitations

The number of non-Newtonian fluid models available to study complex flows is still limited. Mixing studies performed using non-Newtonian suspensions are even less available in the fluid mechanics or physics literature. We present a non-Newtonian suspension fluid system that can be used to mimic real shear thinning suspensions frequently found in biotechnology and food processing applications (e.g., yogurt manufacture, mycelial cell culture, wastewater treatment scenarios). Moreover, in some of these applications, the microorganisms under culture produce and release metabolic products that lower or increase the pH value. Frequently, the pH needs to be controlled by the addition of acidic or basic solutions. The effectiveness of the strategies to control the pH of the culture medium strongly depends on the mixing conditions, and particularly on the proper selection of the acid/base injection point.

Interestingly, blue maize suspensions not only undergo changes in color as the pH value changes, but also in viscosity. Depending on the purpose of a mixing experiment, this attribute of the system can be regarded either as an important disadvantage or a useful property. As pH affects fluid rheology, the measuring attribute (variation of pH in time and space after a pH disturbance in a given location) is bound to affect the flow field of the system under investigation, and therefore the quantity that was meant to be measured. As a consequence, the measurements obtained by acid/base injections are closely linked to the actual disturbance story, which depends on the coupled pH/flow-field dynamics. Consequently, if the purpose of the experiment is to analyze the process of dispersion of an inert tracer, the range of variation in pH during the experiment has to be carefully selected to avoid regimes where the rheology is significantly modified by pH.

Figure 3b and 3c shows the dependence of the apparent viscosity of a blue maize suspension (50% w/w solids) with respect to strain rate at six different pH values. Viscosity is a moderate to weak function of pH at high strain rate values (high fluid velocity values). At medium to low strain rates (Figure 3a), viscosity exhibits a moderate dependence on pH in the range of pH values from 1.6 to 8.0. Above pH 8.0, the dependence of apparent viscosity on pH is significant. Conveniently, as previously discussed, the pH range from 1.6 to 10.6 is also suitable for the evaluation of mixing evolution based on the calculation of color distances in the CIE*Lab* color space. Therefore, in this pH range (1.6–10.6), a variety of mixing experiments can be designed to study different mixing aspects. For example, in an initially weakly basic environment (pH 7.0 or 8.0), acid injection experiments will allow the study of mixing evolution to acidic mixing states (pH 3.3–1.6). This pH range, from 8.0 to 3.0, is relevant in many biological systems. Moreover, this type of experiment allows a good resolution for evaluation of “color distances” in a system in which rheology is practically independent of pH.

The fact that the rheology of this system is a strong function of pH also makes it a versatile model for studying certain aspects of mixing in non-Newtonian fluids. Conveniently, in experiments run at constant pH, the viscosity of blue maize suspensions can be tuned (by adjusting the pH) to mimic non-Newtonian liquids of different viscosities. Moreover, many Non-Newtonian fluids do exhibit a rheology dependence on pH. Examples include yogurt [46]; carbopol solutions [47]; pectin hydrogels [48]; the gums and starch suspensions widely used in food technology applications[49]; protein suspensions of animal origin [50]; poly(acrylic acid) microgels [51]; newly designed surfactants with customized architectures [52], [53]; and even physiologically relevant fluids such as the gastric mucin [54].

As we have discussed before, in biotechnology and food processing applications, the basic or acid injection is frequently used to control the pH of the suspension. In such scenarios, an important aspect to consider is the injection location. As illustrated before (see Figure 4d), experiments can be designed using the blue maize suspension system to investigate the adequacy of an injection location. For example, consider an experiment such that the initial pH of the suspension is set in an acidic value (i.e., 1.6–3.3) and the final mixing point is set around pH 7.0 or 8.0. Injection of a basic solution will allow the pH evolution to the final acidic environment to be followed. If the basic pulse is not effectively dispersed due to the selection of a low circulation injection location, a high pH/high viscosity spot will develop and will be easily visible.

## Conclusions and Final Remarks

In this work, we propose the use of blue maize suspensions as a model to study mixing in Non-Newtonian suspensions. The injection of pulses of acid or basic solutions was used to reveal mixing patterns and flow structures and to follow their time evolution. No foreign pH indicator was required, since blue maize naturally contains anthocyanins that act as a native, wide spectrum pH indicator. We describe a novel method to quantitate mixedness and mixing evolution through Dynamic Color Analysis (DCA). Color readings corresponding to different times and locations within the mixing vessel were taken with a digital camera (or a colorimeter) and translated to the Hunter (or *Lab*) scale of colors using commercial image analysis software. In the *Lab* scale, a 3D color space, each color can be represented by a point, and the difference between two colors can be determined as a distance between two points. We demonstrate the use of distances in the *Lab* scale between a particular mixing state and the final mixing point to characterize segregation or mixing in the blue maize system.

Although a rigorous extension to other non-Newtonian systems cannot be directly made, in this contribution we propose a simple, illustrative, and realistic model for studying mixing in non-Newtonian scenarios. The advantages of this system include the possibility of rheology tuning through pH changes, or the use of acid-base injections to visually assess homogeneity and mixing evolution in time without the need of a foreign pH indicator.

## Materials and Methods

### The fluid model

A suspension of blue maize (BM) flour in water was used as liquid model. In our experiments we used BM kindly provided by Edomex (México). To prepare the suspension, 585 g of blue maize flour were dispersed in 1300 mL of distilled water at 500 RPM in a 3 L fully instrumented Applikon model EZ-control reactor (Applikon Biotechnology, Schiedam, the Netherlands). Curves of apparent viscosity versus strain rate in blue maize suspensions, adjusted to different pH values using NaOH and HCl, were obtained using an automatic Rheometer (Physica MCR 101, Anton Paar, Austria). After pH adjustment, samples were equilibrated at room temperature for 15 min, and then agitated with a spatula to homogenize them. For the determinations reported here, the cone and plate geometry (diameter 5 mm, angle 1°) was used. The gap between cone and plate was set to 1 mm. Flow curves were determined using a steady-state flow ramp in the range of shear rate from 1 to 20 s^−1^ and 20 to 1000 s^−1^. The shear rate was measured point and point with consecutive 20 s steps of constant shear rate. The viscosity was recorded for each point to obtain the flow curves. All measurements were performed in triplicate and average data was reported. An Ostwald-de Waele power-law model [η = K (γ)^n–1^], commonly used to model flour formulations [29]–[31], was used to describe the rheology of blue maize suspensions at different pH values. K and n were calculated from linear regressions of the type ln η = ln K+(n–1) ln (γ).

### Stirred tank system

We used an instrumented stirred tank bioreactor (Applikon, Netherlands) with a working volume of 1.0 to 1.7 L, equipped with a variable speed and pH control system (Figure 1a). The agitator system consisted in a 45° inclined disc impeller [36], [37]. The tank lid was modified to allow for the displacement of the shaft to eccentric positions (if needed), and ports for sampling and dispensing of materials were placed at different angular coordinates, as shown in Figure 1b.

### Visualization experiments

The 3D mixing patterns within the reactor were revealed using basic injections on an initially acidic environment. A low pH initial state was established by adding a hydrochloric acid solution (3N HCl) dropwise at the tank surface until the pH value reached a set point of 4.5. From the initial acidic condition, the excursion of pH induced by injection of a sodium hydroxide solution (1N NaOH) allowed to visualize the advance of the mixing process. For this purpose, the pH control system was activated establishing a new pH set value of 8.5.

The evolution of the mixing process was followed using two different strategies. In the first strategy, frontal photographic images of the entire tank volume were taken at different times using a professional digital camera (Canon Rebel XTi) mounted in a tripod. Special care was taken to maintain an approximately constant illumination quality and camera position at the different time-shots. Images were used to analyze the color evolution within different points of the vessel (Figure 2b). Images were analyzed using the application Color Companion 4.0 for iPad from Digital Media Interactive LCC, USA. In the second strategy, samples from five different locations within the tank volume were taken with a 30 cm pipet at different time points. Samples of 3–5 mL were dispensed in 6-well transparent cell culture plates (Corning, USA). Photographic images of these samples were taken using a professional digital camera (Canon Rebel XTi) mounted in a tripod (Figure 2c and 2d). The color of these samples was determined by image analysis using the application Color Companion 4.0 for iPad from Digital Media Interactive LCC, USA. Alternatively, a colorimeter (Chroma Meter CR-300 from Minolta, NJ, USA) was used to determine color at each well. The color reading was taken by placing the colorimeter exactly underneath each well.

In the experiments used to illustrate the concept of characterization of mixing progress using distances in a color space (Figure 5, 6, 8, and 9) we consistently worked in the range of pH values between 1.6 and 10.6. In the experiments used to reveal mixing pathologies (Figure 4) we did not strictly control the pH variation range.
